# Repetitive transcranial magnetic stimulation over the posterior parietal cortex improves functional recovery in nonresponsive patients: A crossover, randomized, double-blind, sham-controlled study

**DOI:** 10.3389/fneur.2023.1059789

**Published:** 2023-02-16

**Authors:** Chengwei Xu, Wanchun Wu, Xiaochun Zheng, Qimei Liang, Xiyan Huang, Haili Zhong, Qiuyi Xiao, Yue Lan, Yang Bai, Qiuyou Xie

**Affiliations:** ^1^Department of Rehabilitation Medicine, Joint Research Centre for Disorders of Consciousness, Zhujiang Hospital of Southern Medical University, Guangzhou, China; ^2^Department of Rehabilitation Medicine, The First Affiliated Hospital of Nanchang University, Nanchang, China; ^3^School of Basic Medical Sciences, Hangzhou Normal University, Hangzhou, China

**Keywords:** repetitive transcranial magnetic stimulation, disorders of consciousness, unresponsive wakefulness syndrome/vegetative state, randomized control trial, EEG

## Abstract

**Background:**

Recent studies have shown that patients with disorders of consciousness (DoC) can benefit from repetitive transcranial magnetic stimulation (rTMS) therapy. The posterior parietal cortex (PPC) is becoming increasingly important in neuroscience research and clinical treatment for DoC as it plays a crucial role in the formation of human consciousness. However, the effect of rTMS on the PPC in improving consciousness recovery remains to be studied.

**Method:**

We conducted a crossover, randomized, double-blind, sham-controlled clinical study to assess the efficacy and safety of 10 Hz rTMS over the left PPC in unresponsive patients. Twenty patients with unresponsive wakefulness syndrome were recruited. The participants were randomly divided into two groups: one group received active rTMS treatment for 10 consecutive days (*n* = 10) and the other group received sham treatment for the same period (*n* = 10). After a 10-day washout period, the groups crossed over and received the opposite treatment. The rTMS protocol involved the delivery of 2000 pulses/day at a frequency of 10 Hz, targeting the left PPC (P3 electrode sites) at 90% of the resting motor threshold. The primary outcome measure was the JFK Coma Recovery Scele-Revised (CRS-R), and evaluations were conducted blindly. EEG power spectrum assessments were also conducted simultaneously before and after each stage of the intervention.

**Result:**

rTMS-active treatment resulted in a significant improvement in the CRS-R total score (*F* = 8.443, *p* = 0.009) and the relative alpha power (*F* = 11.166, *p* = 0.004) compared to sham treatment. Furthermore, 8 out of 20 patients classified as rTMS responders showed improvement and evolved to a minimally conscious state (MCS) as a result of active rTMS. The relative alpha power also significantly improved in responders (*F* = 26.372, *p* = 0.002) but not in non-responders (*F* = 0.704, *p* = 0.421). No adverse effects related to rTMS were reported in the study.

**Conclusions:**

This study suggests that 10 Hz rTMS over the left PPC can significantly improve functional recovery in unresponsive patients with DoC, with no reported side effects.

**Clinical trial registration:**

www.ClinicalTrials.gov, identifier: NCT05187000.

## Introduction

Disorders of consciousness (DoC) resulting from severe brain injury are among the most challenging conditions encountered in clinical practice (1). They encompass a wide spectrum of conditions ranging from coma to vegetative stage/unresponsive wakefulness syndrome (VS/UWS) (2) to minimally conscious state (MCS) (3). Patients with VS/UWS exhibit reflexive behavior and are unable to perceive themselves or their surroundings (4). In contrast, MCS is characterized by the presence of non-reflexive, cortex-mediated behavior, and there is limited but discernible evidence of self-awareness or environmental awareness (5, 6). The long-term hospitalization of these patients leads to a significant increase in treatment costs, which places enormous pressure on individuals and society in terms of both economic and emotional suffering and raises a host of ethical and legal issues (7). Currently, the available treatments for patients with DoC are limited. However, neuromodulation technology, a non-pharmacological treatment, has been successfully applied to various neurological and psychiatric conditions and holds promise for the treatment of DoC (8).

Repetitive transcranial magnetic stimulation (rTMS) is a noninvasive brain stimulation technique (NIBS) for the human brain. Compared to other NIBS, rTMS can be combined with neuronavigation to excite or inhibit some specific cerebral cortex areas of the brain below the coil (such as the M1 area) (9, 10). Similarly, it has a natural advantage in exploring more complicated domains of other cerebral functions (11). Recently, the rTMS guideline (12) has identified rTMS treatments as having Level A or B clinical evidence for neuropathic pain, depression, the post-acute stage of stroke, and Parkinson's motor function, proving that rTMS can modulate cortical excitability.

Several studies have successfully applied rTMS to treat patients with DoC in recent years. Most studies selected the intervention target of the left dorsal lateral prefrontal cortex (DLPFC). They believed that stimulating the DLPFC can strengthen thalamocortical and cortico-cortical connections and improve behavioral performance, EEG power spectrum, and estradiol levels, particularly in patients in MCS (13–20). However, according to Integrated Information Theory (IIT), consciousness is connected primarily with the posterior cortical areas (21), of which the posterior parietal cortex (PPC) has been demonstrated as the most critical consciousness-associative cortical region (22). It includes the superior marginal gyrus, the angular gyrus, and the precuneus, and it plays a key role in sensory and motor integration and is involved in various cognitive functions (23). Lin et al. (24) found that 14 sessions of rTMS treatment on the bilateral PPC improved clinical scores in one patient in MCS. Meanwhile, EEG and fMRI showed that the directional transfer function (DTF) of the posterior gamma band was significantly increased, and the activity of the inferior parietal lobule was recovered. Legostaeva et al. (25) applied 20 Hz rTMS on the left angular gyrus to 38 patients with DoC and showed improvement in the total CRS-R score in patients in MCS. Auditory and verbal scores improved the most, but there were no effects in patients in VS/UWS. Taken together, neuromodulation with rTMS is a promising way to regulate cortical activity and promote the recovery of behavioral consciousness in patients in MCS, but the effect is unclear for patients in VS/UWS (26), and further pertinent research is needed.

What is consciousness? What are the neuronal correlates of consciousness (NCC)? When scientists registered brain activity in healthy people using a magnetic scanner, they found some active cortical regions, collectively known as “the posterior hot zone” (27). These regions are located in the parietal, occipital, and temporal regions of the posterior cortex and play a crucial role in making up human consciousness. However, significant progress still needs to be made in identifying the true nature of the NCC. Patients with DoC provide a natural model for studying human consciousness. Recent studies revealed that structural and functional connectivity in the default mode network (DMN) correlates with the level of behavioral responsiveness in patients with DoC (28, 29). Decreased activation in the cortical (the middle frontal gyrus and the angular gyrus) and subcortical regions (the thalamus, the cingulate gyrus, and the caudate nucleus) has been observed in patients with DoC, especially in the DMN (30) and the frontal-parietal network (FPN) (31) areas. Furthermore, functional connectivity and structural integrity in the DMN are proportionally related to the index of conscious behavior, especially the posterior cingulate cortex (PCC)/precuneus, which are significantly correlated with the consciousness level and prognosis in patients with DoC (28–30, 32, 33). A cross-sectional study with 72 patients in VS/UWS and 36 patients in MCS indicated that DMN functional connectivity strength decreased in those in VS/UWS compared to those in MCS and positively correlated with CRS-R (34). It was also found that DMN activity was relatively preserved in a small subset of patients in VS/UWS, who eventually evolved to MCS. Therein, the PPC is an important hub of the DMN that plays a central role in multisensory integration (35), environmental-spatial cognition (36), various forms of high-order non-spatial cognition, and so on (37). Furthermore, the PPC is located on the surface of the precuneus cortex near the skull and thus would be an ideal target for rTMS.

Currently, rTMS can increase awareness levels in patients with DoC (38). However, the published results were based on a small sample size or pilot studies (8, 12, 39). In this study, we propose a crossover, randomized, double-blind, sham-controlled rTMS treatment study that uses the left PPC (P3 electrode site) as the target for an intervention program for patients in VS/UWS. CRS-R and EEG were used to evaluate the treatment effects.

## Materials and methods

### Patients

A total of 24 patients in VS/UWS were recruited from the Department of Rehabilitation Medicine, Zhujiang Hospital of Southern Medical University (SMU), Guangzhou, China from November 2021 to July 2022. All patients met the following inclusion criteria: (1) patients aged between 18 and 70 years with acquired brain injuries < 1 year and more than 28 days in VS/UWS; (2) patients with no medical history of neuropsychiatric diseases; (3) patients who have not used any sedatives or other drugs that might interfere with brain stimulation, such as Na^+^ or Ca^2+^ channel blockers or NMDA receptor antagonists; (4) patients with a stable state of disease and vital signs; (5) voluntary agreement given by the families of the patients for the patient's participation in this study with signed informed consent provided; and (6) MRI used to verify the integrity of the left PPC. The exclusion criteria were as follows: (1) patients in other noninvasive or invasive neuroregulation trials; (2) patients with uncontrolled epilepsy or seizure within 4 weeks before enrollment; and (3) patients with contraindications for rTMS or EEG, such as metallic implants in the skull, pacemakers, craniotomies under the stimulated site, and implanted brain devices.

### Study design

**Figure 1:**
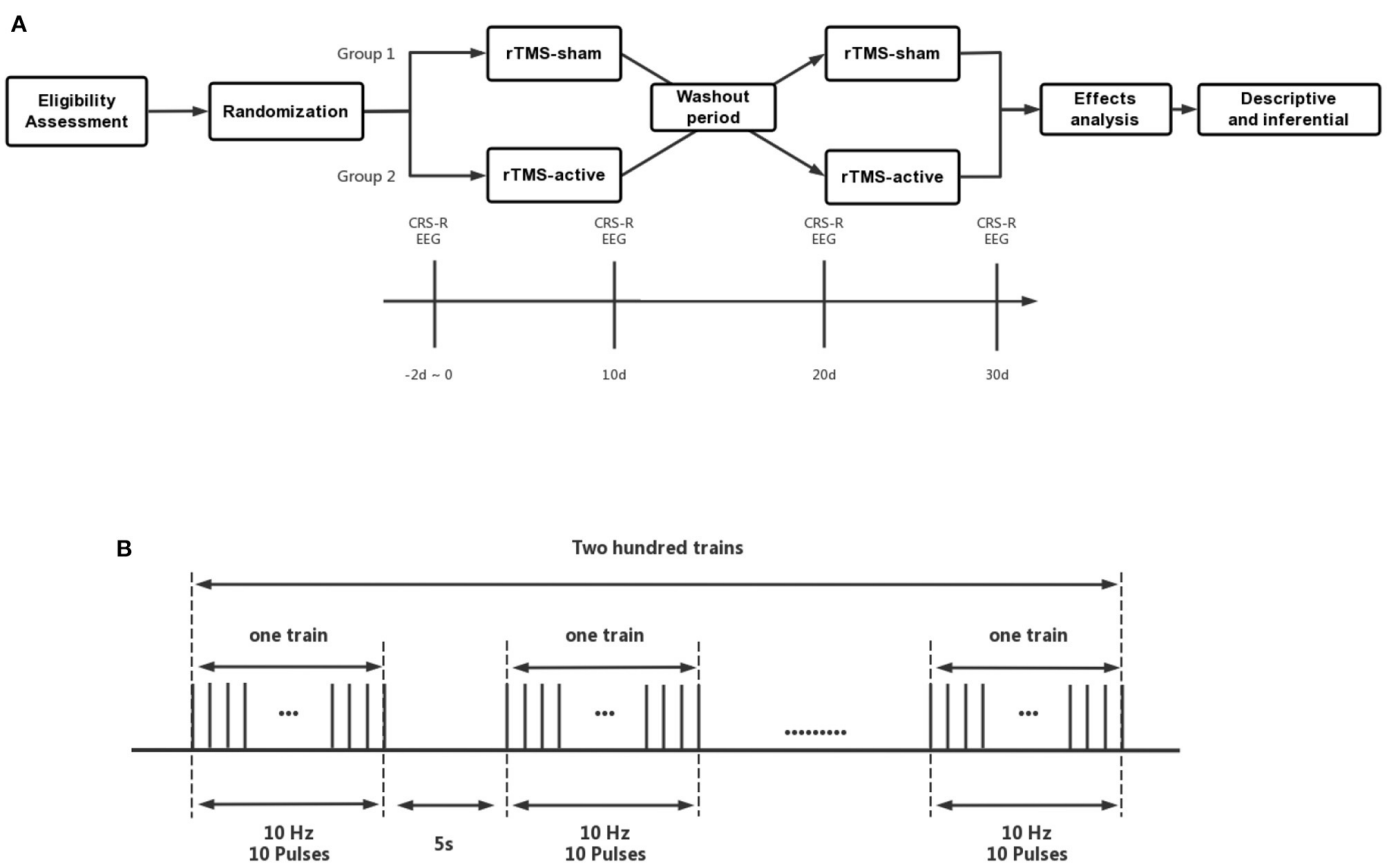
**(A)** The crossover, randomized, double-blind, sham-controlled study protocol, **(B)** Details of rTMS parameters. CRS-R, Coma Recovery Scale-Revised; EEG, Electroencephalogram; rTMS-a, rTMS-active; rTMS-s, rTMS-sham.

This study employed a crossover, randomized, double-blind, sham-controlled design. Participants received 10 sessions of intervention with 10 Hz rTMS-active targeting the left PPC and 10 sessions of rTMS-sham. Ten days' washout period was set between active and sham treatment (Figure 1A). CRS-R (40) total scores after two-stage treatments were considered the primary efficacy outcome. EEG relative spectral power was used as the secondary efficacy outcome.

The study was registered with ClinicalTrials.gov (NCT05187000) and approved by the Ethical Committee of the Zhujiang Hospital of SMU. Patients or their legal guardians who signed informed consent forms (ICF) followed the Declaration of Helsinki. In clinical research, we fully considered the unique characteristics of patients with DoC and their families, such as autonomy, respect for people, and informed consent (41).

### Randomization, blinding, and allocation

Before the baseline period, patients were recruited and divided into two groups in a 1:1 ratio according to computer-generated randomization using the Random Numbers Function of the statistical software SPSS 23.0 (IBM, USA). Randomization was performed blindly by one staff member working under the control of the Data Monitoring Committee (DMC) of Zhujiang Hospital. He was the only person allowed to manage the electronic coding of the randomization to assign the individuals. All patients were assigned a code which was hidden from the allocation process to ensure proper blinding. To perform the allocation concealment process, the blind-coded groups were placed in a closed, opaque envelope and kept by DMC staff. It was opened only during the time of allocation. Both patients and clinic staff (researchers, outcome assessors, caregivers, nurses, physical therapists, statistical analysts, etc.) remained blind to group allocation. The study did not disclose whether the intervention was rTMS-active or rTMS-sham. The rTMS coil was wrapped in a white, opaque plastic paper and labeled as A and B. The rTMS physical therapist (responsible for administering the intervention) was not aware of the group allocation and was instructed by the DMC staff to use Surface A or B first.

### rTMS procedures

Across the experiment, stimulation intensity varied and was determined by the resting motor threshold (RMT), which is defined as the minimum intensity of TMS applied to the M1 region. It could evoke electromyography (EMG) with an amplitude of >50 μV peak-to-peak in the hands' relaxed first dorsal interosseous muscle in more than five out of 10 pulses. The researchers were trained to use the coil surface which was positioned at a tangent angle of 45° to the scalp (42) over the left PPC of the patient to perform rTMS interventions. The rTMS pulses were delivered using an NTK-TMS-II300 stimulator with an IIB502 97-mm figure-of-eight coil (surface A sents active pulses, while surface B sents sham pulses). There were two identical surfaces in this coil; one output rTMS-active pluses, and the other output rTMS-sham pluses (Brain Modulation Technology Development CO, LDT, JiangXi, CHN). A biphasic waveform with a pulse width of ~0.32 ms would be produced.

During the active stage of rTMS treatment, patients received 10 consecutive sessions (one session daily) of stimulation. They were seated in a semi-reclined position on either an ABS bed or a wheelchair, and each session lasted 20 min with a frequency of 10 Hz, delivered over the left PPC (train duration: 1s; inter-train interval: 5s; 200 effective stimulation series; 2,000 pulses at 90% of RMT). An EEG cap marked with the international 10–20 positioning system was used to identify the P3 (left PPc) stimulation site. The rTMS treatment was administered in accordance with safety guidelines (43) (Figure 1B).

During the sham stage of rTMS, patients received 10 consecutive sessions (one session daily) of stimulation. The sham coil was designed to mimic the appearance of the active coil; however, it did not produce a magnetic field and delivered only noise and vibration to mimic the feedback of the active coil. The sham coil was used to control for the placebo effect (44).

### Behavioral assessment

CRS-R (45), as a generally accepted standard, is widely used to define the level of consciousness and assess neurobehavioral recovery in patients with DoC (1). In this study, CRS-R was evaluated by two experienced physicians at four time points: before and after the treatment of the first rTMS stage, after the washout period, and after the second rTMS stage. The CRS-R assessment was conducted between 3 and 5 pm Beijing time. rTMS responders were defined as patients showing new signs of MCS or EMCS in CRS-R (e.g., visual pursuit, pain location, or functional object use).

### EEG recording and preprocessing

EEG was used to evaluate the brain function of patients with DoC (46). In this study, we collected and analyzed the EEG data of patients with DoC at four time points: before the experiment, after the first rTMS stage, after the washout period, and after the second rTMS stage. EEG was acquired from 66 channels (SynAmps^2TM^ 8500; Neurscan, USA) with positions of the 10–20 International EEG system. The equipment used an Ag/AgCl pin electrode with band-pass filtering at DC to 1,000 Hz in the recorder. The EEG sampling rate was set at 2,500 Hz. During the recording period, electrode impedance was maintained below 5 kΩ. We ensured that patients' eyes remained open during all recordings. We used the standard arousal method for CRS-R whenever the eyes of the patients were closed and suspended the assessment if the eyes remained closed.

Offline analysis was conducted using EEGLAB 14_1_1b, running in a MATLAB environment (version 2016a; Math Works Inc., Natick, Massachusetts, USA). The original EEG data were downsampled to 500 Hz and filtered between 1 and 45 Hz. Then, EEG data were divided into epochs of 10 s with 5 s of overlap for each patient, and the noisy segments were manually removed (no more than 20%). The independent component analysis (ICA) was used to eliminate non-neural activities such as blinking and muscle activation. After analyzing the data, the participants' relative power spectral density (RPSD) was calculated using the selected artifact-free EEG epochs across five frequency bands: δ (1–4 Hz), θ (4–8 Hz), α (8–13 Hz), β (13–30 Hz), and γ (30–45 Hz). The investigators calculated RPSD using offline analysis.

### Basic treatments and routine rehabilitation

Qualified rehabilitation therapists at Zhujiang Hospital of Southern Medical University's Department of Rehabilitation Medicine administered various routine rehabilitation programs, including passive limb range-of-motion training, electrical limb stimulation, barometric therapy, respiratory therapy, swallowing therapy, gastrointestinal rehabilitation, and hyperbaric oxygen therapy.

### Statistical analysis

SPSS 23.0 statistical software was used to analyze the results. All the statistical hypotheses were tested by a two-sided test, with the statistically significant level set at 0.05 and the confidence interval of the parameters set at 95%. The independent samples *t*-test and chi-square test were used to analyze and compare the baseline characteristics and the carryover effect between the two sequences. The main effects comparison between treatments, stages, and subjects were performed by the univariate general linear model ANOVA. Considering the crossover of this study, we assessed the carryover effect (i.e., the effect of the first treatment on the second treatment period) at the baseline of the first and second stages. The difference in baseline (measured CRS-R total scores) between the two periods was calculated separately for each patient in two sequence groups for this purpose. If the carryover effect was not significant at the 0.1 level, the different stages were excluded. EEG data were Ln transformed before analysis.

## Result

**Figure 2:**
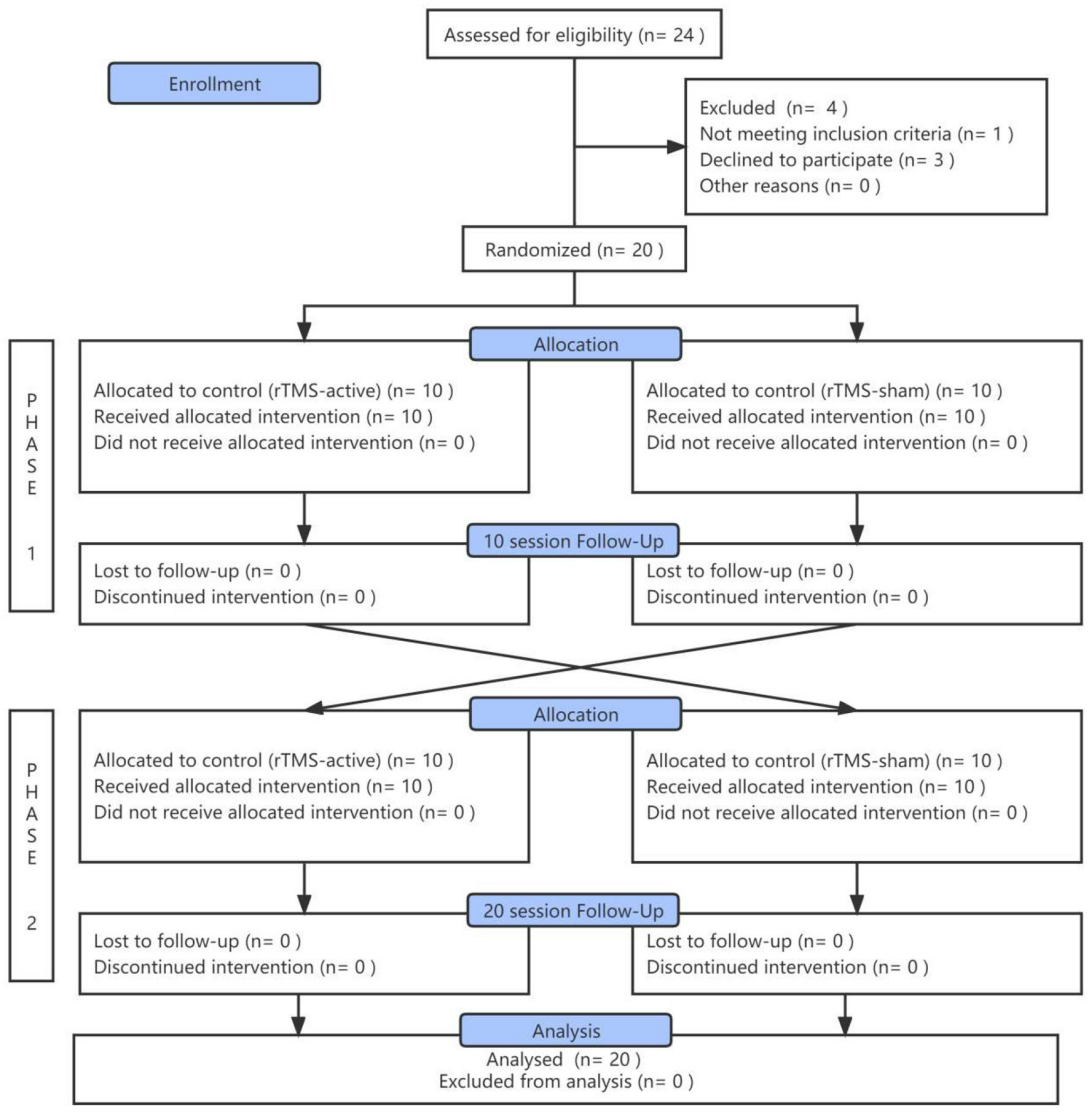
Flow diagram.

A total of 24 inpatients were initially screened; one patient had suffered a stroke, and three patients' family members did not agree to sign the ICF. Twenty patients in VS/UWS completed rTMS treatment successively and were included in the final analysis (Figure 2). Their demographic and clinical characteristics are demonstrated in Table 1. There were no significant differences in age (*t* = −0.574, *p* = 0.573), gender (χ^2^=0.952, *p* = 0.329), time since injury (*t* = −0.142, *p* = 0.944), or baseline CRS-R score (*t* = 0.210, *p* = 0.836) between the two sequence groups (rTMS-active – rTMS-sham vs. rTMS-sham – rTMS-active). There were no adverse events associated with the study.

**Table 1:** Demographic and clinical information of participants.

**ID**	**Age (sex)**	**Etiology**	**Post-injury (months)**	**Treatment Allocation**	**CRS-R presham**	**CRS-R postsham**	**Δ rTMS-s**	**CRS-R preactive**	**CRS-R postactive**	**Δ rTMS-a**	**rTMS responder**
1	34 (M)	TBI	0.9	Sham/Active	7 (1-1-2-1-0-2)	7 (1-1-2-1-0-2)	0	7 (1-1-2-1-0-2)	13 (2-1-6-2-0-2)	6	Responder
2	43(M)	Hemorrhage	11	Active/sham	7 (1-0-2-2-0-2)	7 (1-0-2-2-0-2)	0	7 (1-0-2-2-0-2)	7 (1-0-2-2-0-2)	0	Non-responder
3	40 (M)	HIE	2.2	Sham/Active	7 (1-0-2-2-0-2)	7 (1-0-2-2-0-2)	0	7 (1-0-2-2-0-2)	11 (2-3-2-2-0-2)	4	Responder
4	26 (M)	TBI	1.7	Sham/Active	6 (1-0-2-1-0-2)	6 (1-0-2-1-0-2)	0	6 (1-0-2-1-0-2)	10 (1-3-2-2-0-2)	4	Responder
5	58 (M)	TBI	4.4	Active/sham	9 (1-1-3-2-0-2)	9 (1-1-3-2-0-2)	0	5 (1-0-1-2-0-1)	9 (1-1-3-2-0-2)	4	Responder
6	56 (M)	HIE	1.8	Sham/Active	4 (0-0-1-1-0-2)	8 (2-1-1-2-0-2)	4	8 (2-1-1-2-0-2)	11 (2-3-2-2-0-2)	3	Responder
7	36 (F)	HIE	2.1	Active/sham	6 (1-0-2-1-0-2)	6 (1-0-2-1-0-2)	0	6 (1-0-2-1-0-2)	6 (1-0-2-1-0-2)	0	Non-responder
8	67 (F)	Hemorrhage	2.4	Sham/Active	4 (0-0-2-1-0-1)	5 (1-0-2-1-0-1)	1	5 (1-0-2-1-0-1)	8 (1-3-2-1-0-1)	3	Responder
9	32 (F)	HIE	2.5	Sham/Active	6 (1-0-2-1-0-2)	6 (1-0-2-1-0-2)	0	6 (1-0-2-1-0-2)	6 (1-0-2-1-0-2)	0	Non-responder
10	57 (M)	HIE	2.7	Sham/Active	4 (1-0-0-1-0-2)	4 (1-0-0-1-0-2)	0	4 (1-0-0-1-0-2)	4 (1-0-0-1-0-2)	0	Non-responder
11	66 (F)	HIE	3.6	Sham/Active	7 (1-0-2-2-0-2)	7 (1-0-2-2-0-2)	0	7 (1-0-2-2-0-2)	7 (1-0-2-2-0-2)	0	Non-responder
12	57 (F)	HIE	10.5	Sham/Active	5 (0-0-2-1-0-2)	5 (0-0-2-1-0-2)	0	5 (0-0-2-1-0-2)	5 (0-0-2-1-0-2)	0	Non-responder
13	35 (F)	HIE	2	Active/sham	7 (1-0-2-2-0-2)	7 (1-0-2-2-0-2)	0	7 (1-0-2-2-0-2)	7 (1-0-2-2-0-2)	0	Non-responder
14	67 (M)	Hemorrhage	9.8	Sham/Active	6 (0-0-2-2-0-2)	8 (1-1-2-2-0-2)	2	8 (1-1-2-2-0-2)	7 (1-0-2-2-0-2)	−1	Non-responder
15	43 (M)	TBI	4.5	Active/sham	6 (1-0-2-1-0-2)	5 (1-0-2-1-0-1)	−1	6 (1-0-2-1-0-2)	8 (2-1-2-1-0-2)	2	Non-responder
16	22 (M)	TBI	3.5	Active/sham	7 (1-1-2-1-0-2)	7 (1-1-2-1-0-2)	0	6 (1-1-2-1-0-1)	7 (1-1-2-1-0-2)	1	Non-responder
17	58 (M)	Hemorrhage	3.5	Active/sham	5 (1-0-2-1-0-1)	5 (1-0-2-1-0-1)	0	5 (1-0-2-1-0-1)	5 (1-0-2-1-0-1)	0	Non-responder
18	59 (M)	TBI	2.9	Active/sham	7 (1-1-2-1-0-2)	7 (1-1-2-1-0-2)	0	6 (1-0-2-1-0-2)	9 (1-3-2-1-0-2)	3	Responder
19	61 (M)	TBI	2.1	Active/sham	10 (1-3-2-2-0-2)	10 (1-3-2-2-0-2)	0	8 (1-1-2-2-0-2)	10 (1-3-2-2-0-2)	2	Responder
20	50 (M)	HIE	1.1	Active/sham	4 (0-0-1-1-0-2)	4 (1-0-1-1-0-2)	0	4 (0-0-1-1-0-2)	4 (0-0-1-1-0-2)	0	Non-responder

CRS-R scores are described as follows: Total score (Auditory subscore–Visual subscore–Motor subscore–Oromotor/Verbal subscore–Communication subscore–Arousal subscore); F, Female; M, Male; HIE, hypoxic-ischemic encephalopathy; TBI, Traumatic Brain Injury; CRS-R, Coma Recovery Scale-Revised; Δ, post–pre. In the last column, Responder, patients showing new signs of consciousness after rTMS; Non-responder, patients not showing any new sign of consciousness, taking into account the 4 CRS-R assessments (pre and post-rTMS-active and rTMS-sham) conducted during the study period.

### Primary outcome: Behavioral assessment

The overall CRS-R score showed no significant difference between the first and second stages of treatment (*t* = −0.969, *P* = 0.346). Therefore, the carryover effect was excluded. At the group level, there was a significant rTMS treatment effect (*F* = 8.443, *P* = 0.009). Compared to the rTMS-sham treatment, the rTMS-active treatment exhibited a significant improvement in CRS-R total scores in the patients. The CRS-R details of the univariate general linear model ANOVA are summarized in Table 2.

**Table 2:** Univariate general linear model ANOVA for the CRS-R behavioral results.

**Behavioral results (CRS-R total scores)**					
	**Type III Sum of Squares**	**df**	**Mean Square**	***F***	***P***
Intercept	2016.400	1	.	.	.
Subject	124.700	18	6.928	4.062	0.002
Stage(2)	4.900	1	4.900	2.873	0.107
Treatment(rTMS-s)	14.400	1	14.400	8.443	0.009

Treatment, rTMS-a; rTMS-s, rTMS-sham; stage, 1 (the first period, the other level is 2).

Regarding single subjects, eight patients gained new signs of consciousness following rTMS activation and were defined as rTMS responders. Two patients improved in the motor subscore (functional object use and pain location, respectively), and six patients improved in the visual subscore (visual pursuit). Furthermore, three patients showed improvement in auditory, visual, or arousal functions but did not gain any sign of consciousness. Notably, one patient (P18) gained a visual pursuit after receiving the rTMS-active treatment but lost it in the second stage, only receiving a reserved visual shock. There were no significant differences between responders and non-responders in age (*p* > 0.05), sex (*p* > 0.05), time since injury (*p* > 0.05), or baseline CRS-R score (*p* > 0.05).

### EEG assessment: Relative power and spectral density

**Figure 3:**
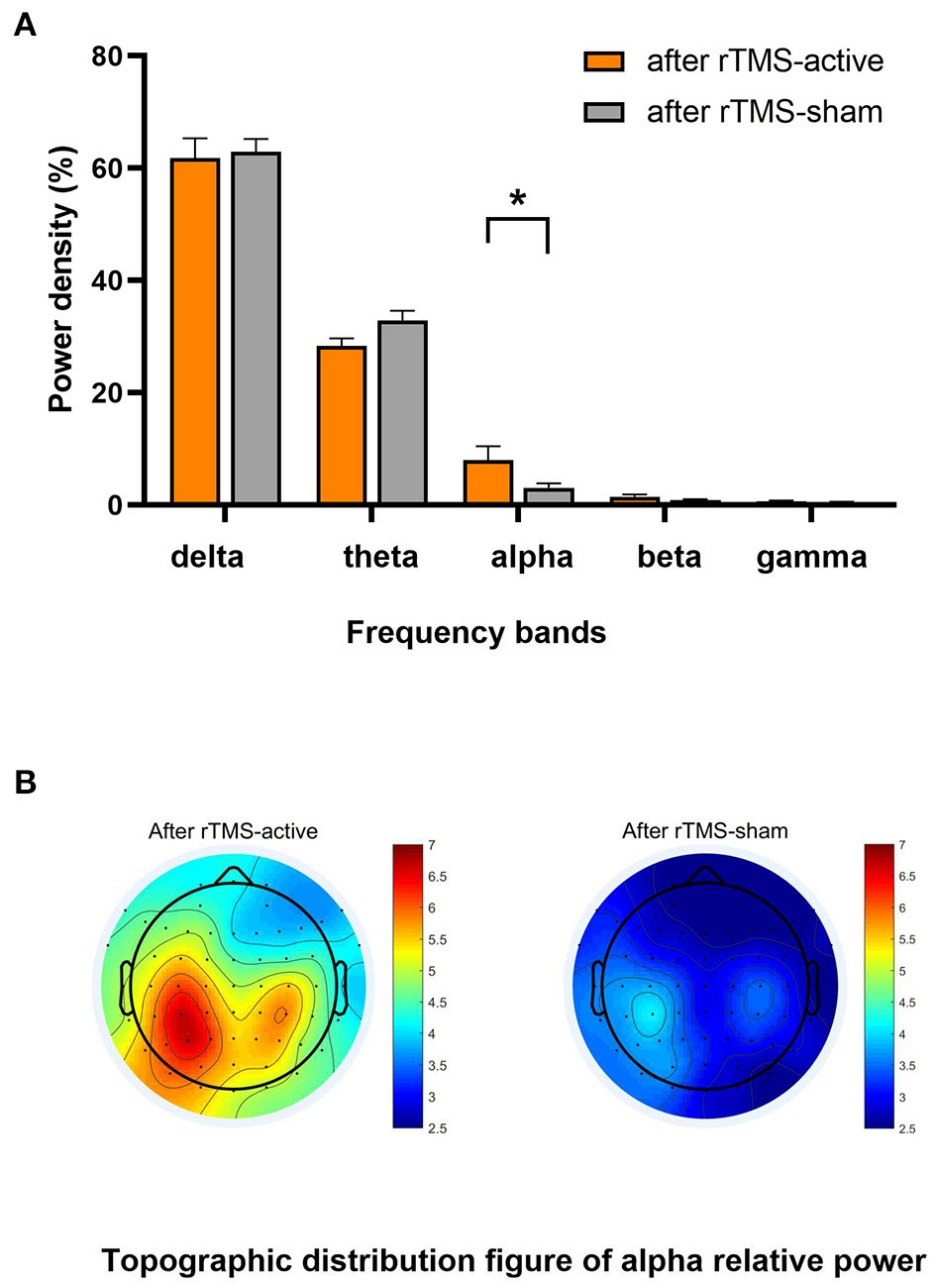
Result of the general linear model ANOVA showed differences in EEG relative power between-group levels (after rTMS-active and after rTMS-sham). **(A)** The relative power in five frequency bands. A statistical significance was only found in the relative alpha power (*p < 0.01). The data were expressed as the means ± SEM. **(B)** There was a difference in whole-brain topographic distribution figure of relative alpha power. Left column: group of after rTMS-active; right column: a group of after rTMS-sham.

**Figure 4:**
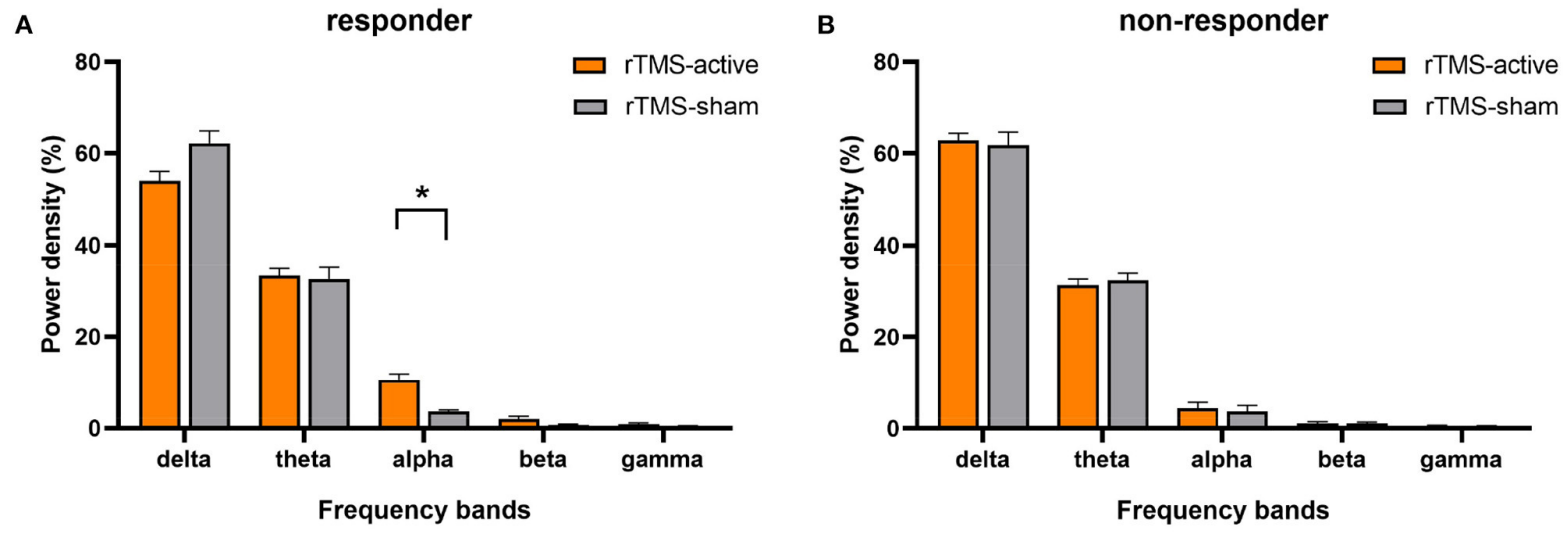
The result of the general linear model ANOVA showed changes in EEG relative power in five frequency bands for responders and non-responders, respectively (rTMS-active and rTMS-sham). **(A)** Statistical significance in responders was found in the relative alpha power (*p < 0.01). The data were expressed as the means ± SEM. **(B)** No significant difference was found for non-responders in any bands.

The univariate general linear model ANOVA revealed that, when compared to rTMS-sham, the rTMS-active treatment demonstrated significantly higher alpha relative power of the whole brain at the group level (*F* = 11.166, *p* = 0.004) (Figures 3A, B; Table 3). For responder patients, the relative alpha power was significantly higher after rTMS-active than after rTMS-sham (*F* = 26.372, *p* = 0.002) (Figure 4A; Table 4). There were no significant differences in non-responder patients (p>0.05) (Figure 4B; Table 4). There were no statistical differences in other bands. We did not observe any evidence for EEG carryover effects or a difference in baseline (see Supplementary materials 1, 2).

**Table 3:** Univariate general linear model ANOVA for EEG relative alpha band power.

**EEG results (Relative Alpha Band Power)**					
	**Type III Sum of Squares**	**df**	**Mean Square**	***F***	***P***
Intercept	40.240	1	.	.	.
Subject	21.929	18	1.218	1.126	0.001
Stage(2)	0.010	1	0.010	0.039	0.845
Treatment(rTMS-s)	2.850	1	2.850	11.166	0.004

Treatment, rTMS-a; rTMS-s, rTMS-sham; stage, 1 (the first period, the other level is 2).

**Table 4:** Univariate general linear model ANOVA for EEG relative alpha band power of responders and non-responders.

**EEG results (Relative Alpha Band Power of responders)**					
	**Type III Sum of Squares**	**df**	**Mean Square**	***F***	***P***
Intercept	13.188	1	.	.	.
Responders	5.542	6	0.924	1.126	0.034
Stage(2)	0.173	1	0.173	1.201	0.367
Treatment(rTMS-s)	4.786	1	4.786	26.372	0.002
**EEG results (Relative Alpha Band Power of non-responders)**					
Intercept	23.919	1	.	.	.
Non-responders	15.797	10	1.580	14.609	0.001
Stage(2)	0.081	1	0.081	0.745	0.408
Treatment(rTMS-s)	0.076	1	0.076	0.704	0.421

Treatment, rTMS-active; rTMS-s, rTMS-sham; stage, 1 (first period, the other level represents 2).

## Discussion

In this crossover, randomized, double-blind, sham-controlled clinical study, we demonstrated the safety, feasibility, behavioral, and electrophysiological effects of using rTMS over the left PPC for the first time in patients in VS/UWS. The crossover design has the advantage of eliminating individual subject differences from the overall treatment effect and is suitable for chronic diseases such as DoC. Therefore, this study's behavioristics and EEG results make a significant clinical observation, which helps explore the target selection of rTMS (even other NIBS treatments) for DoC and improve its clinical diagnosis and treatment (8).

Safety is one of the most important issues of rTMS clinical treatment, especially for seizures. Past literature has reported 20 Hz rTMS-induced seizures in patients with DoC (47). Many situations or complications can contribute to the risk of seizures, such as metabolic abnormalities, fever, and sleep deprivation, which are common in patients with DoC (48). Given that the risk of seizures increases with higher frequency stimulation, our study chose a 10 Hz, 90% RMT stimulus, which is in line with the latest evidence-based guidelines (12) and safety guidelines (43), to ensure the safety of the treatment while effectively activating the target area. As expected, there were no adverse events related to rTMS by the end of the study. This study not only demonstrated the feasibility of this protocol in patients in VS/UWS but also showed the effectiveness of rTMS in combination with other rehabilitation techniques (passive limb range-of-motion training, swallowing therapy, hyperbaric oxygen therapy, etc.).

In this study, our primary results demonstrated for the first time that the left PPC is a highly promising rTMS target for improving functional recovery in unresponsive patients. Compared to rTMS-sham, the CRS-R total score at the rTMS-active level increased significantly, suggesting that rTMS above the left PPC increases awareness levels in unresponsive patients. It shows that the left PPC is a key hub of the DMN, and increasing its activity plays a crucial role in the recovery of consciousness (49). Among these patients, eight were rTMS responders; seven progressed into MCS (4 TBI, 2 HIE, 1, and hemorrhage) after being rTMS-active, and one entered EMCS (P1, TBI). The CRS-R subscales showed that these responders regained consciousness at the visual and motor levels (six visual pursuits, one functional object use, and one pain location), which is consistent with the improvement of subscale items in responders in former studies of rTMS for DoC (14, 17, 25, 50). Our results may indicate that the residual expression of consciousness is more preserved in the visual and motor pathways in unresponsive patients (51, 52). This is consistent with a recent study that found that the regulation of PPC plays an important role in the alerting and maintenance of visuospatial attention (27), as well as in the recovery of consciousness. Thus, we need to devote more attention to observational and intervention studies in this field in the future. It is crucial to aid in the functional recovery of patients with DoC and establish a correct prognosis (53, 54).

EEGs, which provide objective, widely applicable, direct, and immediate information, are essential in DoC research (55). Compared with patients with MCS, patients with VS/UWS have decreased alpha power (56). The improvement of alpha and its source power as a prognostic measure in the parieto-occipital lobe is closely associated with the probability of consciousness recovery in patients with VS/UWS (57). Specifically, in patients with a DoC of < 1 year, alpha power and its variability are vital predictors for functional recovery (26). In healthy adults, EEG activity during the awake resting state is typically dominated by the alpha rhythm, which is distinct from that of patients with disorders of consciousness (DoC) (58). Our findings support this conclusion: compared to rTMS-sham, the relative alpha power was increased after ten sessions of rTMS-active treatments, particularly at the left PPC stimulation target. Furthermore, eight responders had significantly higher relative alpha power after rTMS-active at the group level, but there was no significant change in non-responders. This suggests that the increase in relative alpha power may be a signature of response to 10 Hz rTMS in responders and may also be a characteristic of covert consciousness in unresponsive patients. Overall, the EEG analysis in this study supports the conclusion that 10 Hz rTMS over the left PPC may improve brain function.

Due to the brain's sensitivity to ischemia and hypoxia, patients with DoC and HIE who suffer from cardiac arrest (CA) usually have a poor prognosis (40). In the study by Legostaeva et al., no change was observed after rTMS treatment in the VS/UWS subgroup. This may be due to the fact that the majority of patients (93%) are caused by HIE. Previous research showed that only 16.1% of patients in VS/UWS caused by HIE respond to rTMS treatment (8). A recent study that used a single session of rTMS for patients with DoC and HIE did not observe any behavioral or EEG changes and suggested that rTMS should not be recommended for these patients (18). However, in this study, two of the eight patients with HIE (25.0%) progressed from VS/UWS to MCS after treatment (P3 and P6). For a patient whose P3 stimulation site was caused by electrical damage, gender was male, and the time since injury was 2 months, the CRS-R score improved to MCS (1-3-2-1-0-2) and relative alpha power significantly increased after 10 sessions of rTMS treatment. For a patient whose P6 stimulation site was caused by CA lasting for a minute, gender was male, time since injury was 3 months, the CRS-R score improved to MCS (2-3-2-2-0-2) and the relative alpha power significantly increased after 10 sessions of rTMS treatment. This suggests that patients with HIE still have the opportunity to recover consciousness from VS/UWS with timely and continuous rTMS treatment.

In addition, we have another important consideration. A growing body of literature indicated that the misdiagnosis rates remain high (30–40%) (59, 60). Some patients with residual consciousness are considered to be unresponsive (59, 61), suggesting that some patients in VS/UWS may be in MCS or may even be fully conscious (62, 63), such as with cognitive motor dissociation (CMD) (64) or locked-in syndrome (LIS) (65). In our study, two experienced physicians evaluated CRS-R two times to determine the patient's level of consciousness and to reduce the rate of misdiagnosis during the eligibility assessment stage. However, we still have to acknowledge the limitation that the methods currently available, such as behavioral tests and task-free or task-based measures for DoC, cannot provide evidence for the complete absence of consciousness (66). Once a patient has been clinically diagnosed to be in a VS/UWS, those possible errors can result in a poor prognosis and ineffective decision-making (61). They will not have the chance to receive active treatment, which may lead to the withdrawal of water and food (i.e., the termination of life support) (67). This can be a tragedy for their families. As treatments for patients in VS/UWS are currently limited, our results suggest that 10 sessions of rTMS should be used for nonresponsive patients with or without covert functional activities of consciousness. As a diagnostic treatment, it may be more significant for nonresponsive patients than neural measures. This is why we focused on nonresponsive patients in this study.

However, there are still several limitations to this study. First, we did not use rTMS combined with MRI navigation technology but instead used the P3 electrode of the 10–20 international EEG system to locate the left PPC, which cannot ensure precise locations of the stimulus. This method is more clinical as it is less expensive and less complicated, and there are fewer hospitals and institutions equipped with a navigation system. Therefore, our results can provide direct guidance for rTMS treatment for patients with DoC. Second, there are relatively few objective evaluation methods used in this study. Future studies should focus on TMS-evoked potential (TEP), perturbational complexity index (PCI) (68), or EEG source localization analysis induced by TMS-EEG (69).

Theta burst stimulation (TBS) is a new form of TMS in which rapid bursts of 50 Hz are delivered within slow-wave theta (5Hz) oscillations (70). Recently, TBS has been increasingly used as a therapeutic intervention for psychiatric and neurologic diseases (71). Wu et al., in their exploratory study, used intermittent thetic-burst stimulation (iTBS) over the left DLPFC in eight patients with DoC, of which seven of them showed an increased CRS-R score and increased EEG power of alpha (15). Compared to traditional rTMS, the biggest advantage of TBS is that completing its standard stimulation protocol only takes 3 min and it has a lower stimulation pulse intensity (72, 73). This not only saves time for patients' clinical treatment but also improves patients' compliance and increases treatment quality. In short, TBS is a promising avenue for DoC research in the future.

In conclusion, this crossover, randomized, double-blind, sham-controlled clinical study provides new evidence for the clinical application of rTMS in patients with VS/UWS. The results indicate that 10 Hz rTMS on the left PPC can improve functional recovery and significantly increase the relative alpha power of the whole brain, indicating that the treatment may be potentially considered to assist in the timely recovery of consciousness.

## Data availability statement

The raw data supporting the conclusions of this article will be made available by the authors, without undue reservation.

## Ethics statement

The studies involving human participants were reviewed and approved by Ethical Committee of Zhujiang Hospital of Southern Medical University. The patients/participants provided their written informed consent to participate in this study.

## Author contributions

CX, YB, and QXe conceived and designed the study protocol and contributed to the draft of the manuscript. CX, WW, and XZ wrote the manuscript and participated in the coordination and implementation of the study. YB and QXe revised the study protocol and wrote several sections of the manuscript. XH, QL, QXa, HZ, and YL helped develop the study measures and data collection. All authors contributed to the manuscript's draft and approved the final manuscript.
