# Supplementary figures and images for: Repetitive transcranial magnetic stimulation over the posterior parietal cortex improves functional recovery in nonresponsive patients: A crossover, randomized, double-blind, sham-controlled study
Source: Front Neurol. 2023 Feb 16;14:1059789. doi: 10.3389/fneur.2023.1059789 (PMC9978157; doi:10.3389/fneur.2023.1059789)

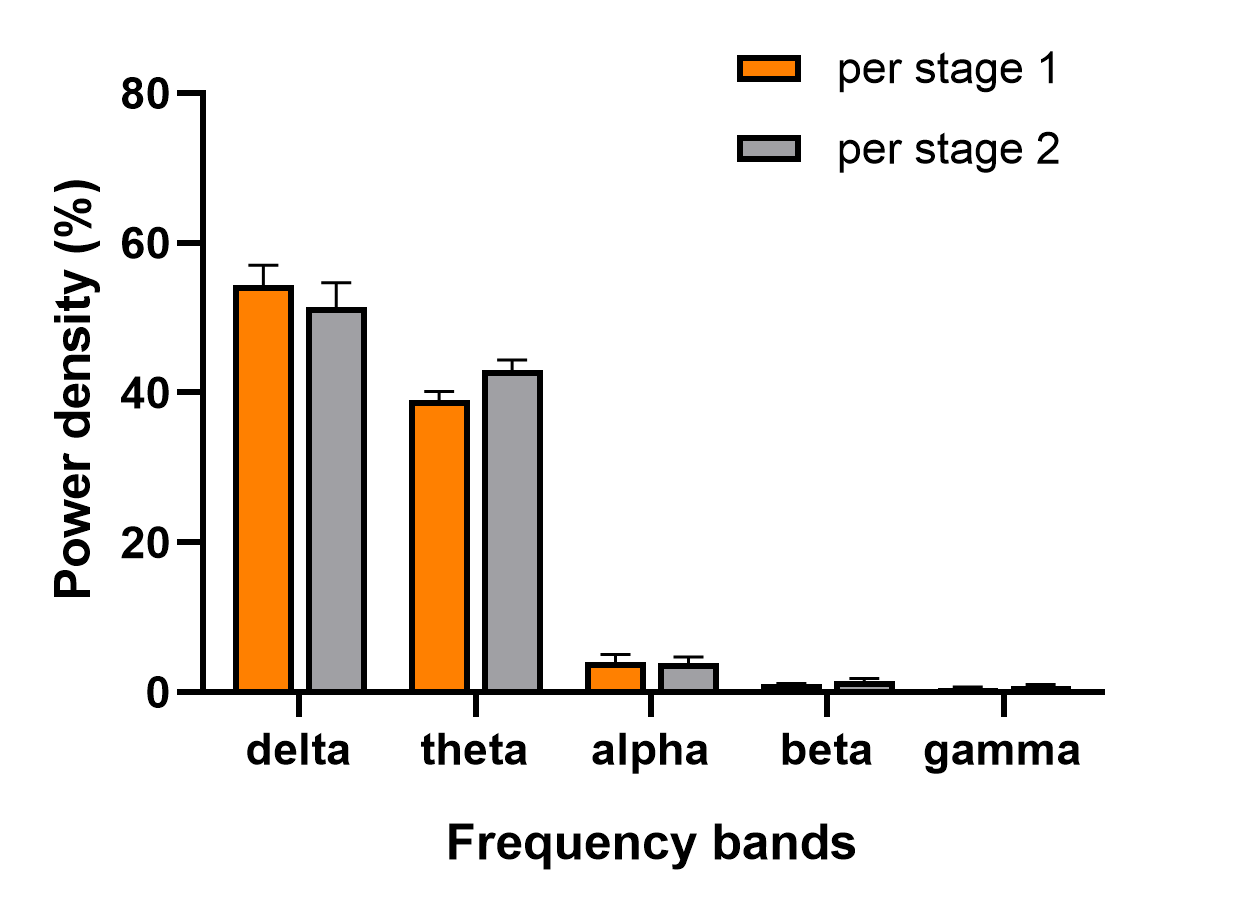

Supplement: Supplementary file 1 [file Image_1.TIF]

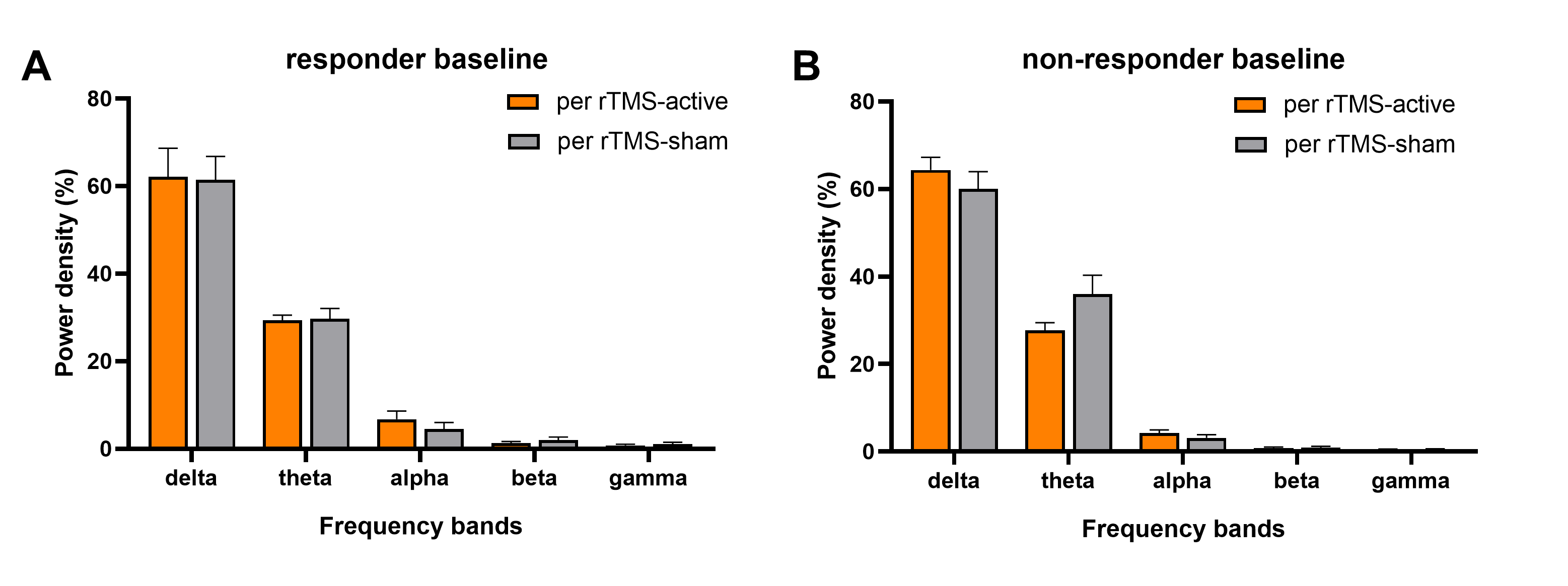

Supplement: Supplementary file 2 [file Image_2.TIF]
